# Clinical impact of COVID-19 in patients with carbapenem-resistant *Acinetobacter baumannii* bacteraemia

**DOI:** 10.1017/S0950268823001644

**Published:** 2023-10-10

**Authors:** Jeong Yeon Kim, Woo Joo Lee, Jin Woong Suh, Sun Bean Kim, Jang Wook Sohn, Young Kyung Yoon

**Affiliations:** Division of Infectious Diseases, Department of Internal Medicine, Korea University College of Medicine, Seoul, Republic of Korea

**Keywords:** *Acinetobacter baumannii*, carbapenem resistance, coronavirus disease 2019, mortality, SARS-CoV-2

## Abstract

The aim of this study was to evaluate the impact of coronavirus disease 2019 (COVID-19) on treatment outcomes in critically ill patients with carbapenem-resistant *Acinetobacter baumannii* (CRAB) bloodstream infection (BSI). This single-centre, retrospective cohort study was conducted in a 1,048-bed university-affiliated tertiary hospital in the Republic of Korea from January 2021 to March 2022. The study participants included consecutive hospitalised adult patients (aged ≥18 years) in the intensive care unit with CRAB monomicrobial BSI. During the study period, a total of 70 patients were included in our study, and 24 (34.3%) were diagnosed with COVID-19. The 28-day mortality rate was 64.3%. In the multivariate Cox proportional hazard regression analysis, diagnosis of COVID-19 (hazard ratio (HR), 2.91; 95% confidence interval (CI): 1.45–5.87), neutropenia (HR, 2.76; 95% CI: 1.04–7.29), Pitt bacteraemia score (per point; HR, 1.30; 95% CI: 1.19–1.41), and appropriate definite antibiotic therapy (HR, 0.31; 95% CI: 0.15–0.62) were independent predictors of 28-day mortality in patients with CRAB BSI. In conclusion, our findings suggested that COVID-19 has a negative prognostic impact on patients with CRAB BSI. Further study is needed to investigate the specific mechanisms of how COVID-19 worsens the prognosis of CRAB infection.

The coronavirus disease 2019 (COVID-19) pandemic has caused significant changes in public health, including an increase in infections caused by multidrug-resistant organisms (MDROs) [1]. Limited treatment facilities in negative pressure isolation rooms, as well as a lack of resources, may have contributed to the negative impact on MDRO prevention and control practices. Previous studies have reported that patients with severe COVID-19 and secondary bloodstream infection (BSI) present with more critical conditions, and with higher mortality rates [2, 3]. Particularly, COVID-19 was a significant predictor of mortality among patients with BSI caused by multidrug‑resistant Gram-negative bacteria [4]. However, detailed data on specific pathogens remain limited.

Carbapenem-resistant *Acinetobacter baumannii* (CRAB) is a challenging nosocomial pathogen in patients admitted to intensive care units (ICUs) and is suggested as one of the most prevalent organisms causing opportunist infections in patients with COVID-19 [5]. An antimicrobial resistance surveillance system in South Korea reported that the carbapenem resistance rate of *A. baumannii* strains was as high as 89.9%, and mostly associated with *bla*_OXA-23_-like genes. During the COVID-19 pandemic, we have seen patients with COVID-19 and CRAB BSI progress rapidly to fulminant septic shock. The purpose of this study was to assess the impact of COVID-19 on clinical outcomes in critically ill patients with CRAB BSI. A retrospective cohort study was conducted in a 1,048-bed tertiary hospital in the Republic of Korea from January 2021 to March 2022. The participants were consecutive hospitalised adult patients (aged ≥18 years) in the ICU with CRAB monomicrobial BSI. The study protocol was approved by the Institutional Review Board of Korea University Anam Hospital (approval no. 2022AN0268), and the requirement for written informed consent was waived due to the retrospective nature of the study.

Identification of *A. baumannii* isolates was conducted with matrix-assisted laser desorption/ionisation time-of-flight mass spectrometry (MALDI-TOF MS) (Bruker Diatonic GmbH & Co. KG, Bremen, Germany) and antimicrobial susceptibility of isolates was determined using the Micro Scan Pos Combo Panel Type 6 automated system (Baxter Diagnostics, West Sacramento, CA, USA). A diagnosis of COVID-19 was based on a positive nasopharyngeal swab or sputum specimens tested with real-time reverse transcription-polymerase chain reaction assays. Data on the clinical characteristics and outcomes were obtained by reviewing electronic medical records.

Pearson’s *χ* ^2^ and Fisher’s exact tests were applied for categorical variables, and the Mann–Whitney *U* test for continuous variables, in comparisons between COVID-19 and non-COVID-19 groups among patients with CRAB BSI. To identify risk factors associated with mortality of CRAB BSI, multivariate logistic regression analysis using the backward stepwise variable selection based on likelihood ratio statistic was used. Variables with a *P*-value <0.20 in the univariate Cox proportional hazard regression analysis were included in the multivariate analysis. The level of significance was set at 0.05. Statistical analyses were performed using the IBM SPSS statistics programme (version 23.0; IBM Corporation, Armonk, NY, USA).

In total, 70 patients were included in the study; 24 (34.3%) were diagnosed with CRAB BSI while undergoing COVID-19 treatment in an ICU equipped with a negative pressure facility. A comparison of the demographic and clinical characteristics and outcomes between patients with and without COVID-19 is presented in Table 1. Catheter-related BSI (57.1%) was the most common cause of BSI, followed by primary bacteraemia (31.4%), pneumonia (7.1%), and intra-abdominal infections (5.7%). The median indwelling time of the central venous lines was 7 days (interquartile range (IQR) 3.0–13.5). Patients with COVID-19 had significantly shorter catheter-indwelling time than those without COVID-19 (4 days (IQR 2.25–7.50) vs. 9 days (IQR 5.25–14.75), *P*-value = 0.011). Likewise, patients with COVID-19 were older than those without COVID-19 (Table 1). The time interval from hospital admission to BSI diagnosis was shorter in COVID-19 patients (Table 1). There was no significant difference in the Charlson’s comorbidity scores between the two groups; however, cardiovascular diseases were more common in patients without COVID-19 (Table 1).

**Table 1.** Comparison of demographic and clinical characteristics according to COVID-19 status in patients with bacteraemia caused by multidrug-resistant *Acinetobacter baumannii*

	COVID-19	Non-COVID-19	Total	*P*-value
	(*n* = 24)	(*n* = 46)	(*n* = 70)	
Demographic and clinical characteristics				
Age (years), median (IQR)	75 (66.0–86.3)	69 (52.5–80.0)	71 (61.8–81.0)	0.035^a^
Male, *n* (%)	14 (58.3)	25 (54.3)	39 (55.7)	0.475
Total length of hospital stay (days), median (IQR)	20.5 (12.3–40.3)	33.5 (18.8–74.8)	29.5 (15.0–63.8)	0.025^a^
Admission to the time of MDR-AB bacteraemia (days)	7.5 (4.3–15.0)	14.5 (7.0–29.0)	10.5 (6.0–24.5)	0.032^a^
Pitt bacteraemia score	7 (4.3–14.0)	6 (3.8–14.0)	6.0 (4.0–14.0)	0.42
Underlying conditions				
Charlson’s comorbidity score, median (IQR)	5 (4–6)	5 (4–6)	5 (4–6)	0.985
Hypertension, *n* (%)	16 (66.7)	21 (45.7)	37 (52.9)	0.077
Cardiovascular diseases, *n* (%)	0 (0.0)	9 (19.6)	9 (12.9)	0.017^a^
Malignancy, *n* (%)	4 (16.7)	8 (17.4)	12 (17.1)	0.610
Chronic kidney diseases, *n* (%)	4 (16.7)	10 (21.7)	14 (20.0)	0.433
Operation history within 30 days of MDR-AB bacteraemia	9 (37.5)	34 (73.9)	43 (61.4)	0.003^a^
Neutropenia, *n* (%)	0 (0.0)	8 (17.4)	8 (11.4)	0.028^a^
Immunosuppressants, *n* (%)	23 (95.8)	18 (39.1)	41 (58.6)	<0.001^a^
Prior third-generation cephalosporine usage within 30 days of MDR-AB bacteraemia, *n* (%)	12 (50.0)	12 (26.1)	24 (34.3)	0.042^a^
Appropriate antibiotic treatment, *n* (%)				
Empirical antibiotics within 48 h of MDR-AB bacteraemia	16 (66.7)	32 (69.6)	48 (68.6)	0.505
Definite antibiotics	17 (70.8)	30 (65.2)	47 (67.1)	0.422

COVID-19, coronavirus disease 2019; IQR, interquartile range; MDR-AB, multidrug-resistant *A. baumannii*.

a Statistically significant difference.

Immunosuppressants were administered more frequently to patients with COVID-19 than to those in the negative group (Table 1). Remdesivir, dexamethasone, and anticoagulants were prescribed for the great majority of the 24 patients with COVID-19, and nine received tocilizumab. With the exception of third-generation cephalosporins, exposure to antibiotics within 30 days before the diagnosis of CRAB BSI showed no significant differences between the two groups, and there was no difference in antibiotic treatment for CRAB bacteraemia between the two groups (Table 1). The CRAB BSI-related mortality and 28-day mortality rates were 61.4% and 64.3%, respectively, and with higher rates in patients with COVID-19; however, the difference was not statistically significant (Table 2). The median number of days from the development of bacteraemia to death was numerically lower in patients with COVID-19 (Table 2) and half of the latter group died within 7 days of the bacteraemia event, in contrast to 13 (28.3%) patients without COVID-19 during the same period.

**Table 2.** Comparison of clinical outcomes according to COVID-19 status in patients with bacteraemia caused by multidrug-resistant *Acinetobacter baumannii*

	COVID-19	Non-COVID-19	Total	*P*-value
	(*n* = 24)	(*n* = 46)	(*n* = 70)	
28-day mortality, *n* (%)	18 (75.0)	27 (58.7)	45 (64.3)	0.138
MDR-AB bacteraemia-related mortality, *n* (%)	18 (75.0)	24 (52.2)	42 (61.4)	0.054
Bacteraemia to death (days), median (IQR)	5 (2–14)	12 (2.75–24)	9 (2–23)	0.103
Bacteraemia to death in 7 days, *n* (%)	12 (50.0)	13 (28.3)	25 (35.7)	0.072
Mechanical ventilation, *n* (%)	20 (83.3)	43 (93.5)	63 (90.0)	0.177
Renal replacement therapy, *n* (%)	12 (50.0)	22 (47.8)	34 (48.6)	0.531
ECMO, *n* (%)	4 (16.7)	7 (15.2)	11 (15.7)	0.564
Septic shock, *n* (%)	23 (95.8)	43 (93.5)	66 (94.3)	0.575

COVID-19, coronavirus disease 2019; ECMO, extracorporeal membrane oxygenation; IQR, interquartile range; MDR-AB, multidrug-resistant *A. baumannii.*

Univariate regression analysis using 30 variables was performed to identify prognostic factors associated with mortality. Subsequent multivariate analysis confirmed a diagnosis of COVID-19 (hazard ratio (HR), 2.91; 95% confidence interval (CI): 1.45–5.87), neutropenia (HR, 2.76; 95% CI: 1.04–7.29), and Pitt bacteraemia score (per point; HR, 1.30; 95% CI: 1.19–1.41) to be independent predictors of 28-day mortality in patients with CRAB BSI. Appropriate specific antibiotic therapy (HR, 0.31; 95% CI: 0.15–0.62) was a notable independent predictor of reduced mortality.

To the best of our knowledge, this is the first study to analyse the clinical impact of COVID-19 on mortality in patients with CRAB BSI. Our findings demonstrate that such patients showed a significantly high mortality rate (64.3%) during the pandemic, and co-infection with COVID-19 was associated with an increased risk of 28-day mortality.

Critically ill patients with COVID-19 admitted to the ICU and treated with prolonged mechanical ventilation are associated with an increased risk of secondary infections. Accordingly, several CRAB outbreaks have been reported in these patients worldwide and *A. baumannii* has been a leading agent of bacterial co-infection in COVID-19 patients [6]. Our study focused on patients with CRAB BSI, in contrast to others which included various presentations of CRAB infection including BSI [5, 7]. In line with our results, the latter studies also observed a significant role of CRAB co-infection in the mortality rate of those with COVID-19, compared to other clinical presentations [5, 7]. Indeed, a recent study has also suggested that COVID-19 has a negative prognostic impact on patients with multidrug-resistant-Gram-negative BSI [3].

We found catheter-related BSI (57.1%) to be the most common cause of CRAB BSI, followed by primary bacteraemia (31.4%). This is in accord with a previous study which suggested that COVID-19 patients were 3.5 times more likely to develop BSI compared to a negative cohort, with a significant increase between pre-pandemic and pandemic nosocomial BSI rates [8]. These findings may be reflective of a higher degree of illness in patients with severe COVID-19. However, other factors that contribute to the risk of secondary infections should be considered together as the majority of patients with severe COVID-19 are often prescribed corticosteroids, other immunomodulators, and broad-spectrum antibiotics, and are exposed to invasive devices and long-term stays in the ICUs. In keeping with the foregoing study [8], our results also showed significant immunosuppressant administration in COVID-19 patients. Immune dysregulation caused by the virus and immunosuppressant administration may possibly be associated with worsening outcomes of CRAB BSI in these patients. However, the requirement of specific clinical facilities for COVID-19 patients may be a limitation of our study.

During the repeated pandemic surges, infection prevention practices may have been affected by the exhaustion of healthcare workers, low compliance with infection control measures, shortage of isolation rooms, and inadequate antibiotics routinely prescribed to patients with COVID-19. The rapid expansion of the ICU for the management of such patients could also have contributed to increased nosocomial infection rates and prevalence of CRAB in the hospital environment [6]. Here, antibiotics were prescribed empirically to 95.8% of patients with COVID-19 to avoid bacterial co-infections or superinfections, which is comparable to the antibiotic prescription rates (70%) in a previous study [9]. Therefore, the vulnerability of this population to healthcare-associated infections should be underscored for early diagnosis and intensive infection control measures for CRAB BSI, and other opportunist bacterial agents.

Neutropenia, appropriateness of antibiotic treatment, and Pitt bacteraemia score were identified as key predictors of mortality in patients with CRAB BSI, in addition to COVID-19 co-infection. Clinical severity of bacteraemia and appropriate antimicrobial therapy are widely recognised as predictors of mortality, and neutropenia should also be considered as another characteristic of clinical predisposition and/or severity of bacteraemia in COVID-19 patients [10].

This study had some limitations. First, it was of a retrospective design and confined to a single centre, and along with the relatively small sample size, unrecognised clinical factors may have contributed biases in the data. Second, molecular analysis of CRAB isolates was not performed to evaluate the impact of clonal or virulence factors on clinical outcomes. Although MALDI-TOF MS is suitable for identifying *Acinetobacter* species which would be encountered in a clinical setting, it is recognised that *A. baumannii* is a complex comprising a number of named species which require specific molecular techniques for their differentiation, clonal relatedness, and pathogenic potential [11]. Nevertheless, our investigation study is, to the best of our knowledge, the first study to evaluate the clinical outcomes of COVID-19 co-infection in patients with CRAB BSI.

In conclusion, patients hospitalised with COVID-19 who developed CRAB BSI were significantly more likely to die than those without COVID-19. As there is no optimal treatment for CRAB infection, preventive measures such as active infection control and antibiotic stewardship are top priorities. With the COVID-19 pandemic changing the epidemiological features of CRAB infection, it is necessary to verify our findings in a large-scale study and investigate the specific mechanisms of how COVID-19 worsens the prognosis of CRAB infection.

## Data Availability

Raw data for this study are available upon request with the corresponding author.
